# Challenges in the execution of public health research: Reflections from Public Health Research Initiative (PHRI) grant management in India

**DOI:** 10.1016/j.dialog.2022.100020

**Published:** 2022-05-30

**Authors:** Deepak Saxena, Poonam Trivedi, Ruchi Bhatt, Sandul Yasobant, Priya Bhavsar, Khushi Kansara, Farjana Memon, Dileep Mavalankar

**Affiliations:** a Department of Public Health Science, Indian Institute of Public Health Gandhinagar (IIPHG), Gandhinagar 382042, India; b School of Epidemiology & Public Health, Datta Meghe Institute of Medical Sciences (DMIMS), Wardha 442004, India; c Center for One Health Education, Research, and Development (COHERD), Indian Institute of Public Health Gandhinagar (IIPHG), 382042 Gandhinagar, India

**Keywords:** PHRI, SERB, grant management, public health grants, India

## Abstract

**Background:**

Well-planned health research is fundamental to the success of any public health system in leading to better population health outcomes. Although the Indian public health system is unique, it lacks strong linkages between research and practice. There is a pressing need to address the gap in the research to reduce the disease burden in the country. Although various efforts are made to enhance public health research, such research is rarely documented as a process. The objective of the present paper is to document issues and challenges in managing public health research grants awarded to the PHRI fellows from 2013-to 2021 under the PHRI project.

**Method:**

A mixed-method approach, including qualitative (in-depth) interviews and secondary review, was adopted to collect the challenges in executing PHRI grants (during 2013–2021). The in-depth interviews were conducted among the PHRI execution team, whereas the secondary document review was conducted among the PHRI fellows, and the findings are documented under major themes like administrative, technical, and financial issues and/or challenges.

**Result:**

A total of 35 candidates 16 intramural (IM) candidates affiliated with PHFI or IIPH institutes and 19 extramural (EM) candidates affiliated to other academic institutes were selected for the fellowship, The common challenges identified amongst intra & extramural fellows were inability to disseminate the study findings, challenges in communication and getting audited statements, changes in study methods without prior permission, mid study attrition of CO-PIs and high budget utilization. The specific difficulties identified from extramural fellows were change in institute affiliation, lack of support to fund utilization from the parent institute and difficulties in field validation.

**Conclusion:**

The present perspective emphasizes that the management and implementation of a research grant is the crucial part of achieving a project's desired outcome. The learnings of PHRI grant execution allows the researchers to understand the issues in terms of methodological rigour and financial guidelines, rigorous tracking of the project activities, and complying with the terms of funding agreement are crucial. The challenges explored in this grant execution recommend developing a structured public health grant management leadership program for researchers and executors.

## 1 Introduction

Despite evidence-based policy research and a continued drive from both policymakers and researchers to increase research uptake in policy, barriers to the use of evidence are persistently identified in the literature [1,2]. Thus, building research capacity is essential for strengthening the healthcare system in low and middle-income countries [3,4]. When research capacity is limited, there is a void in the production of contextually relevant research and the synthesis of research evidence to inform practice, programs, and policies [4,5]. Although research is increasingly recognized as one of the dynamic forces behind global health and development, the research output from low- and middle-income countries such as India compares poorly with high-income countries [6]. Quality research output from India is grossly lacking, and there is relatively little research output in several diseases/ conditions that cause a significant disease burden in India [7]. A study conducted by Dandona et al. revealed that many leading causes of disease burden across communicable, non-communicable diseases, injuries, and other health priorities continued to be underrepresented in public health research output [8].

These continuing shortfalls in public health research need to be addressed to reduce the enormous disease burden in India. Hence, to provide effective stewardship for capacity building in research, the Science and Engineering Research Board (SERB), a statutory body under the Department of Science and Technology (DST), collaborated with the Public Health Foundation of India (PHFI) to establish the Public Health Research Initiative (PHRI) [9]. This initiative was set up to fund Indian researchers at selected institutes focused on public health, as per an established process (discussed in detail later). SERB implemented the initiative with techno-managerial implementation support from PHFI,Indian Institute of Public Health, Gandhinagar (IIPHG), a constituent unit of the PHFI, dealt with the technical part of the project [10].

Research and innovation are growing in India, with significant investments being made towards institutions, researchers, and research infrastructure [7]. Multiple fellowships have been offered at a national and global scale for capacity building in public health research; however, the difficulties faced during public health research grant management are poorly researched. The challenges faced during the implementation of public health research can provide useful learnings to grant managers of the future fellowship program. It can also provide the base for the preparation of policies for effective grant management.

This perspective aims to document issues and challenges in managing public health research grants awarded to India's PHRI fellows during 2013–2021. This study can provide a learning opportunity for the future grant management team to plan for execution and to maximize impact.

## 2 Material and methods

This perspective adopted a mixed-methods approach, including qualitative (in-depth) interviews and secondary review. As part of the qualitative methods, in-depth interviews of various stakeholders such as Technical Advisory Group (TAG) members (03), PHRI research managers (01), grant managers/ executors and coordinators(02), were interviewed during 2013–2021. These interviews were taken at the various location during the grant meetings/ workshops. Two of the trained qualitative researchers were conducted these interviews in english, and the recording was documented with their consent. All the interviews were transcribed for the analysis. A thematic analysis was conducted based on the groundedness of the codes. As part of the secondary review, the progress reports and the financial statements of the PHRI fellows were reviewed by the PHRI team members.

### 2.1 Data synthesis

The in-depth interviews were analyzed, and major issues faced by the grant management team were documented. The challenges faced during the management of each grant were also documented from the review of progress reports, financial reports, and findings from the field visits by the PHRI team.

## 3 Results

### 3.1 PHRI grant overview

**Fig. 1:**
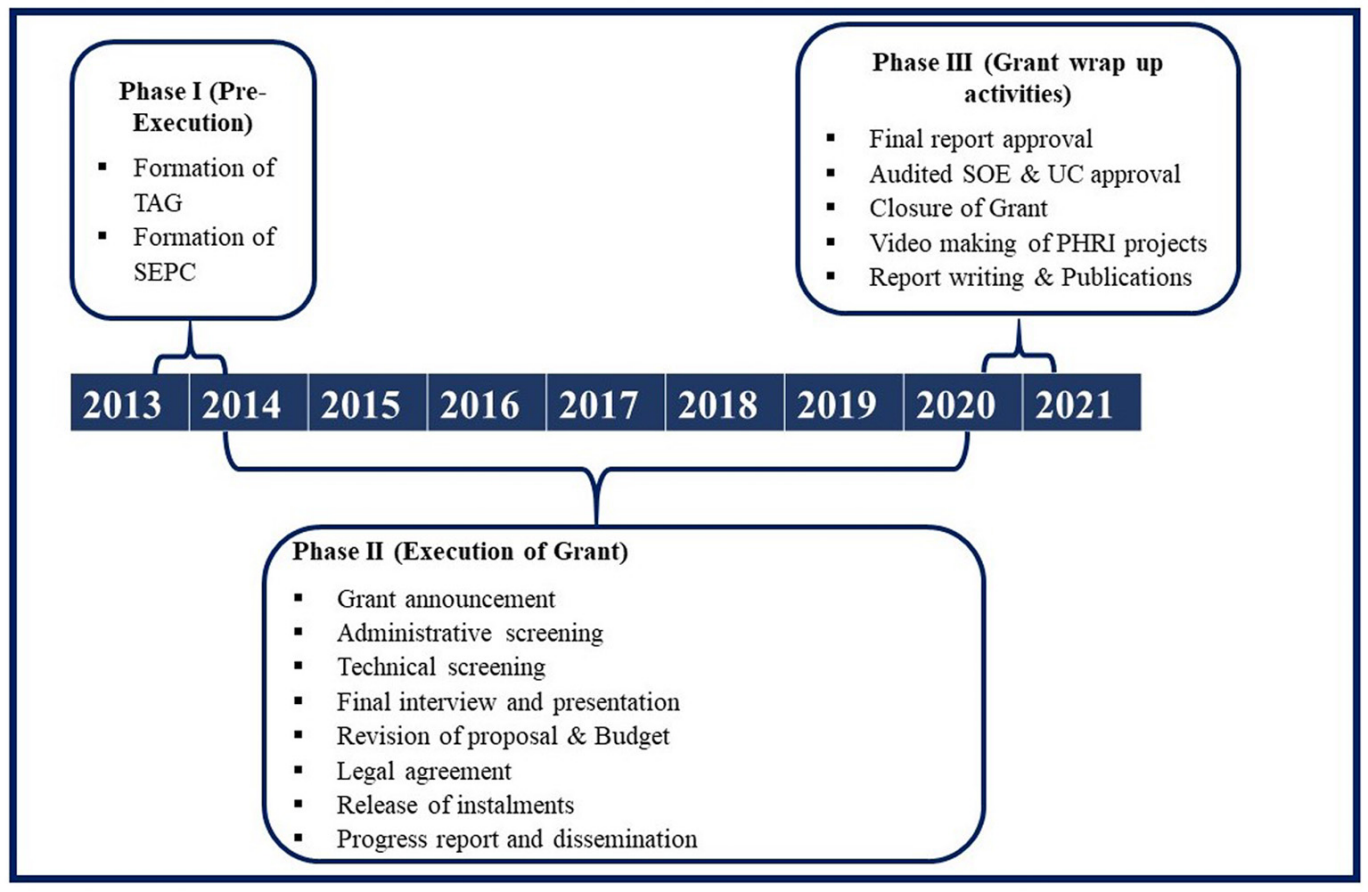
Stepwise process of PHRI grant management during 2013–2021 in India.

The life cycle of the PHRI grant consists of three major phases, a) pre-execution (2013–14), b) execution (2014–20), and c) post-execution (2020–21) (Fig. 1).” During the pre-execution phase for the operational management of grants, the TAG was formed to provide oversight in research projects. The PHRI grants involve intramural support to the fellows within PHFI/IIPHs and extramural support to fellows from other Indian institutes. IIPHG established a Standing External Peer Committee (SEPC) chaired by the IIPHG director to ensure transparency. The committee comprises 12 senior experts from various academic institutions from multiple disciplines. This committee was responsible for the evaluation of the shortlisted proposals based on the participant's proposal presentation, and the final reports of the successful fellows were also reviewed and approved by SEPC members.

. During the second phase, activities like the announcement of the grant, administrative and technical screening, and a final interview and presentation of the shortlisted candidate were conducted. After reviewing the proposals and budget of selected candidates by the PHRI team and finance department of IIPHG, a legal agreement between the fellow's institute and PHFI was executed. After the execution of an agreement, the funds were transferred to that institute in three installments as per the tearms of the agreement.

In the last phase of post execution, a progress report was sought from the fellows every year to get the financial and project updates. After completing the project, the final report and audited Utilization Certificate (UC) were submitted by the fellows, reviewed and approved by the finance team and SEPC members. A total of three symposiums were also organized where the fellows presented their projects, and SEPC members provided feedback for each project.

### 3.2 PHRI grant distribution

A total of 36 grants, comprising 16 intramural candidates affiliated with PHFI or IIPH institutes and 20 extramural candidates affiliated to other academic institutes, were selected for the PHRI fellowship during four rounds (2014–2019). However, out of 36, one fellow withdrew her application, so a total of 35 grants were executed. During the first round (2014–15), 9 (6 intramural and 3 extramural) fellows were selected (Table 1). During the second round (2015–16), 14 (7 intramural and 7 extramural) and Third round (2017–18), 8 (1 intramural and 7 extramural were selected. In the last round (2018–19), a total of 5 (2 intramural and 3 extramural) candidates were selected.

**Table 1:** Year-wise distribution of granted PHRI fellows during 2013–2021 in India.

Type	1st round (2014–15)	2nd round (2015–16)	3rd round (2016–17)	4th round (2018–19)	Total
Intramural fellows	06	07	01	02	16
Extramural fellows	03	07	07	03	20
Total	09	14	08	05	36

PHRI: Public Health Research Initiative.

**Fig. 2:**
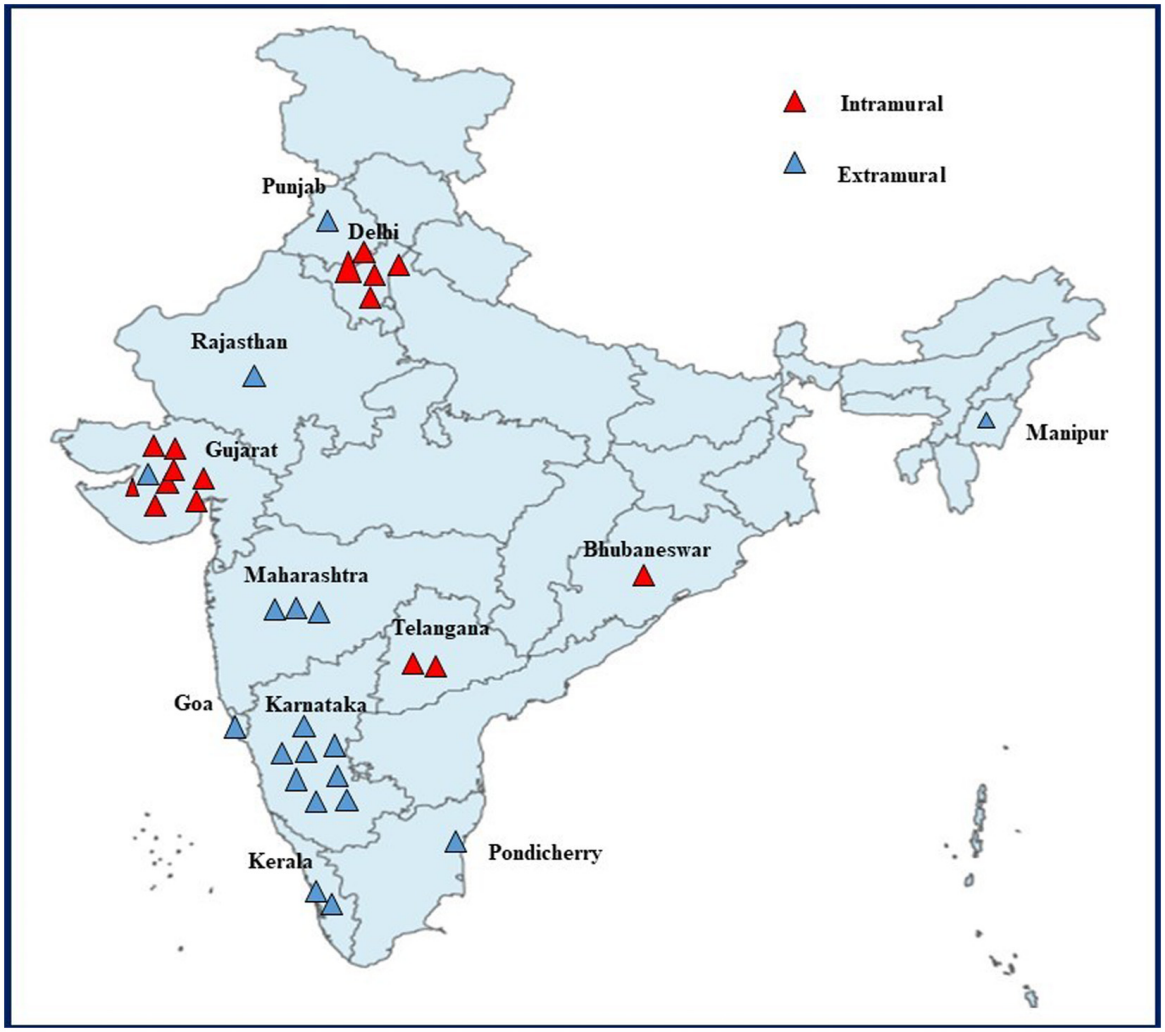
Geographical distribution of granted PHRI projects during 2013–2021 in India.

The selected fellows represented Gujarat, Karnataka, Maharashtra, Delhi, Kerala, Rajasthan, Punjab, Goa, Telangana, Pondicherry, Bhubaneswar, and Manipur (Fig. 2). The fellows were selected in an equal gender ratio. Out of 35 fellows, 18 were males, and 17 were female.

Of 35 projects selected, most (43%) were related to non-communicable diseases (NCD), with 20% being on maternal and child health (MCH) and 23% being on health management and 14% being on infectious diseases (Table 2).

**Table 2:** Theme wise distribution of granted PHRI projects during 2013–2021 in India.

Public health domain	Number of projects n(%)
Non-communicable diseases	15 (43)
Communicable diseases	5 (14)
Maternal & Child Health	7 (20)
Health management	8 (23)
Total	35 (100)

PHRI: Public Health Research Initiative

### 3.3 PHRI grant execution & management challenges

**Fig. 3:**
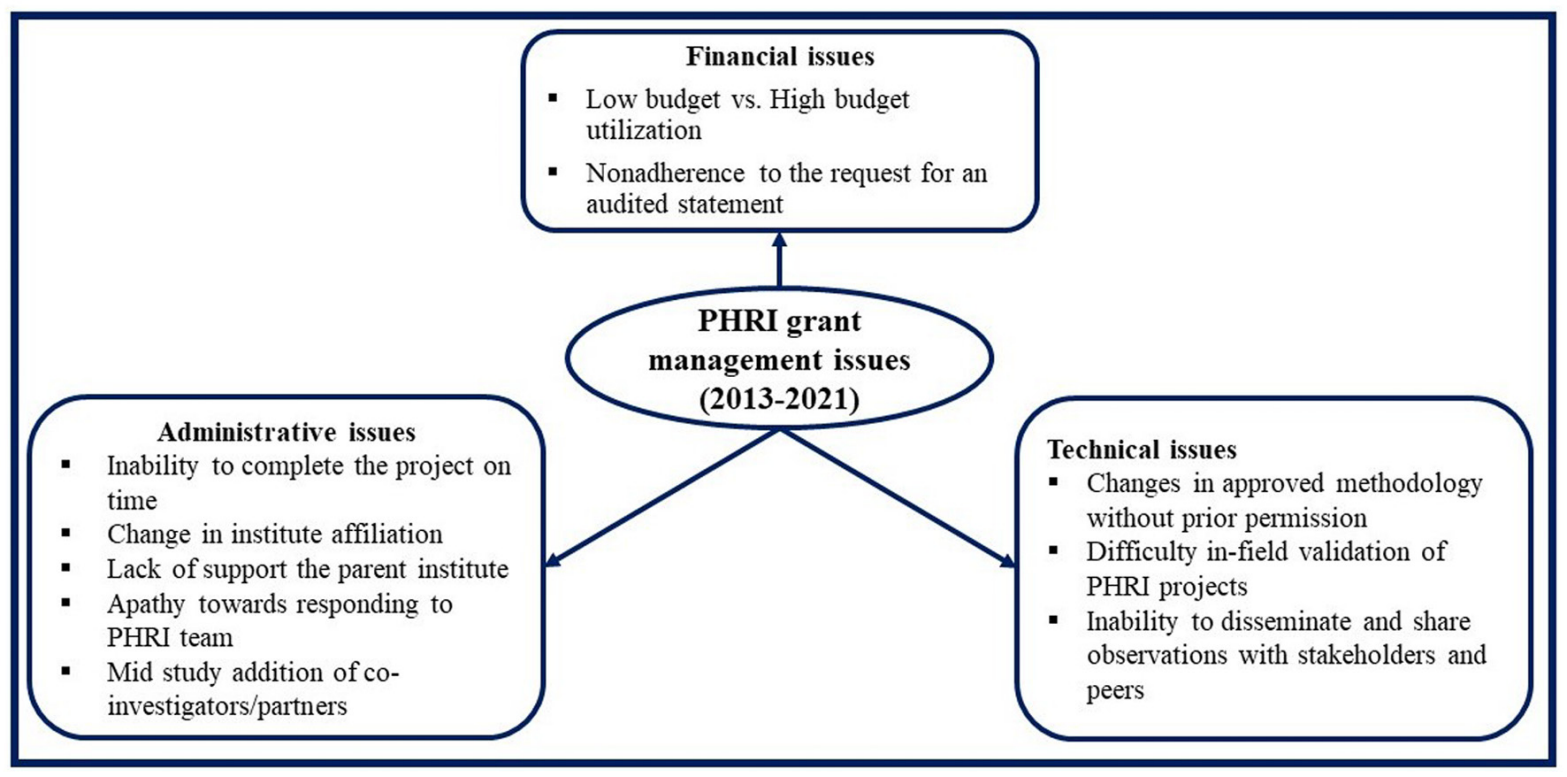
Summarized issues and challenges of PHRI grant management during 2013–2021 in India. PHRI: Public Health Research Initiative.

Based on the documentation of efforts in the execution of grants during 2013–21, grant management challenges are summarized in the main themes of administrative, financial, and technical (Fig. 3).

#### 3.3.1 Administrative issues

##### 3.3.1.1 Inability to complete the project on time

The total duration approved for the grant was a maximum of three years. However, the fellows had proposed a period ranging between 12 and 36 months. Most of the approved project's duration was 24 months. We documented the period in which the fellows completed the grant; unfortunately, all fellows except one had applied for No Cost Extension (NCE). The request for a NCE, mostly for six months, but five fellows (3 intramural, 2 extramural) requested more than two NCEs. The most common reason cited was a delay in the execution of the project activities.

##### 3.3.1.2 Change in institute affiliation (moved to another organization)

A formal agreement between the fellow's institute and grant agency involves many administrative and legal work. However, as the grant duration ranged from 1 to 3 years, three fellows (extramural) had changed their parent institute before signing the agreement. This led to many managerial procedures and undue delay in releasing the subsequent tranche of funds and implementation of the study. Obtaining signed approvals from the legal team, finance team, and head of the grant agency is also a time-consuming and lengthy process.

##### 3.3.1.3 Lack of support for fund utilization from the parent institute of the grant holder

Support from the fellow's host/parent institute is one of the critical factors for successfully implementing the grant project. As per the protocol, the agreement was executed between the funding agency and employing parent institute. After the contract, PHFI transferred funds to the employing parent institute. However, for one of the grant holders (extramural), the parent institute had created hurdles in initiating the study and release of the grant to the grant holder despite adequate funds. PHRI finally transferred the funds back to the funding agency, and a new agreement was made with the co-principal investigator's (Co-PI's) institute. This process leads to substantial delays in the execution of the proposed project. Most grant holders reported that a lot of paperwork needs to be done with the parent institute in releasing the grants. Hence, despite having adequate dedicated funds for PHRI, there was a delay in execution.

##### 3.3.1.4 Challenges in communication

Communication and coordination with the fellows are essential for getting regular updates on the project. The current study found non-adherence of researchers/ fellows to the communication from the funding agency. Most of the fellows' communication was about the release of funds and requests for extension. Otherwise, the fellows did not often communicate to the management team to provide updates and submit financial details. As a part of the capacity building of the fellows, regular workshops were planned and conducted. However, there was a reluctance to attend this workshop. A few fellows were chronic absentees, arguing that they were already aware of the workshop topics or citing other engagements.

For effective grant management, it is essential to take time-to-time updates from the fellows and take the necessary action as appropriate during projects. In the current study, fellows were asked to submit six-monthly progress reports. However, very few fellows submitted reports on time (almost none) without reminders. Many fellows required frequent reminders and calls for submitting a progress report. The reminders ranged from 3 to 24 calls and three to more than a dozen reminder emails.

##### 3.3.1.5 Mid study addition of CO-PIs / Partners

During the proposal submission, it was explicitly requested to share the details of partners and Co-PIs; however, fellows added new Co-PIs without prior information or approval from the PHRI. The team rectified this, and a new advisory was issued with a request to inform team PHRI about any new addition. This was observed for both intra & extramural fellows.

#### 3.3.2 Technical issues

##### 3.3.2.1 Changes/execution in approved methods without prior permission

The TAG accepted the proposal only after a detailed deliberation on methods, and the legal agreement had a clause about asking team PHRI for permission to deviate from the proposed methods. However, the fellows went on changing the methods and study site without seeking permission from the funding agency. This affected the outcome of the study and the financial part of the study, which requires prior approval from the TAG and the finance department. Two fellows have also faced issues related to government permission. E.g., one of the intramural fellows submitted a proposal for three states; however, he could not get permission from one state, so he was required to change the budget. Various deliberations between the funding agency and researchers were needed, leading to delays. Another researcher has to change the study methods due to the inability to get permission. In one case (intramural), the researcher's study was based on a rural area; however, due to travel budget and feasibility, he/she needed to change the study site from rural to urban.

##### 3.3.2.2 Difficulty in-field validation of PHRI projects

As a part of proposal approval, the timeline and the Gantt chart were approved by TAG. One of the inbuilt mechanisms to improve rigour was to validate the data collection process in the field. However, the project field validation was challenging and required frequent discussions with the researchers. After periodic reminders and calls, the researcher finalized the visit to the field. When the team visited the area, they were denied access to the site because of permission issues from the concerned authority. This has been exclusively observed amongst the extramural fellows.

##### 3.3.2.3 Inability to disseminate and share observations with stakeholders and peers

One of the significant issues the PHRI team faced was the fellows' inability to disseminate the observations to relevant stakeholders. It was required from the fellows to submit their research for publication in peer-reviewed journals of academic repute. There was a provision for publication cost for open access journals in the budget and an additional budget. However, reluctance to publish their work was a common observation. PHRI TAG formed an expert faculty group to help fellows publish their studies in scientific journals. To improve the researcher's publication skills, workshops were organized by the team. This had slightly enhanced the publication performance, but team PHRI could still retrieve only 24 publications from the fellows on the approved proposal. While investigating the outcome in the form of publication, only 40% fellows published at least one scientific manuscript during or after completion of the fellowship. The publication number seems to skew some fellows who went on doing more than one publication as per their interest, as it was not a mandate of the grant call. There is little difference between the extramural and intramural fellows in terms of publications. Out of 16 intramurals, 44% published their research in scientific journals and however, amongst the 19 extramutrals only 35% published their research. The papers were published in peer-reviewed journals like Public Health, the Indian Journal of Community Medicine, Medical Science, BMC Public Health, PLOS One, Political Science, etc. The impact factor of these journals varies from 0.1 to 5.715. The impact of these published articles either operationalized with actions or not was out of scope to this analysis and was not documented after the grant period.

#### 3.3.3 Financial issues

##### 3.3.3.1 Low budget vs. high budget utilization

The experience from the current project shows that researchers tend to submit a budget on the higher side; however, at the end of the project, researchers fail to fully utilize the budget. Only four of 35 projects used all the funding as proposed and approved. Hence, the burnout of the remaining 31 projects which could not use all the funding ranged from 30% to 70% of approved funds.

The fund utilization also highlighted one significant issue revealed while dissecting the Statement of Expenditure (SOE) review against the approved budget. Two fellows (one intramural & one extramural) purchased high-end equipment which was not proposed in the approved budget. Prior permission from the funding agency was also not sought. PHRI team saw a similar inter-head transfer (transfer of money from one budget head to another head) of funding beyond the agreed 10% as per the legal agreement in most SOE.

Other observations included repeated requests for revision of the budget. As per the protocol of PHRI, the PHRI team set the original budget within the limit of the maximum grant amount. However, many instances of fellows requesting revised budget costs exceeding the maximum grant amount. There was a deviation from the approved budget regarding inter head allocation, new items, and allocation to new heads. The most common inter-head allocation change was observed in equipment, additional salary cost (which was not permissible), personnel, stationery and printing, and travel. Frequent communication between team PHRI and the researchers was required for adjustment.

The researcher could get 25% of the total budget at the initial phase per protocol. However, almost all researchers requested more than 75% of the total budget to initiate the study citing reasons for purchasing high-end equipment at the initial stage, such as laptops, software, and tablets. The majority of the fellows could not justify the inter head changes and the need for 75% budget allocation.

Few researchers, mostly intramural fellows, requested funds to travel to the states for government permission even before the agreement was executed. Only one of the extramural researchers also asked to pay processing fees for getting Institutional Ethical Clearance (IEC) ahead of the formal release of the grant. In such cases, the PHRI team took formal approval from the TAG to fulfil the request.

##### 3.3.3.2 Challenges getting requested audited statements

To get the next installment, an audited statement of expenditure had to be submitted to the team. Non-adherence to submitting the audited statement was observed, which required frequent communication and discussion with the researchers. A few of them were not aware of the audited report and required auditors' signatures.

## 4 Discussion

The present paper emphasized that writing a good proposal and obtaining a grant are insufficient for a successful grant. Still, management and implementation are the most vital part of getting the desired outcome of the public health research. It is also essential to keep the funding agency informed about the project's status, changes, and challenges in the project. Communication challenges with fellows lead to delays in administrative procedures, which have also been evinced in other blended grant management situations. [11,12]. There is a culture of proposing an excessive budget for the research project, which is then only partly used by the end of the project. Inappropriate implementation and planning of research were also identified as a significant concern, which further delayed the completion of the project and the outcome of the research studies, as also documented in other global research [13,14]. Induction training of researchers in grant management and financial training could solve many issues related to finance management and help the researchers prepare a realistic budget. Project finances must be monitored regularly, and monthly financial summary reports should be discussed with researchers.

The present paper highlights the need for strengthening the research capacity of the public health workforce. Many of the fellows failed to disseminate/ publish their study findings, reflecting a need for strengthening their publication skills. As the fellowship was awarded to individuals and not to the institution, why few fellows failed to publish depends on their interest and experience.

Periodic training on writing good research papers by providing local mentors at the beginning of the fellowship can enhance the skills of fellows. Further, there is a need to document the outcome of such fellowships, which can be measured through publications, building networks with alumni, receiving feedback from the fellows' supervisors, and documenting their career progress after completing the fellowship.

We have collated the issues and challenges of managing 35 research studies implemented in various parts of India. The lessons learned from this perspective can allow better management of future public health research fellowship programs globally and in India. The paper also provides learnings to new researchers to manage various challenges in implementing their research studies and get the desired outcome.

Grant management includes all the administrative responsibilities researchers must complete during the time frame of the grant. When the researcher manages a grant, they must ensure that they keep all the promises made in the proposal, which means staying in compliance with the terms of the grant, following through on all the deliverables, and submitting reports according to the funder's requirements. Mismanaging grants can lead to a range of consequences for an organization, limiting the number of grants one can receive in the future.

Some researchers may need to adapt their proposals because of changed circumstances or challenges in the field. The proposed activities may not be feasible, leading to a mid-study change in methods or site. Ultimately, the outcome of the project suffers. Hence it is essential to carry out a pilot (feasibility, formative) study to get exposure to the field realities. It can also serve as the basis for budget calculation and helps in the preparation of the actual budget required for the project.

Most of the fellows had requested a NCE in the present study, and many even asked for more than two no-cost extensions, which may be due to various reasons. The reasons for the same were not analyzed in the present study. Proper guidelines and policies for tracking fellows' technical and financial activities are essential. Induction training on grant management and administrative requirement and writing a research paper can improve the public health research fellowship outcome.

There are a few limitations of this study. First, none of the fellows were interviewed as part of the data collection, the ideal method would be designing a primary study for collecting the data from each fellow. Second, the replicability of the findings might not be appropriate for the internationally funded projects. As these findings are exclusively a grant from an Indian perspective and managed by an Indian body, it will be not appropriate to other lines of funding. However, the learning will remain an interesting finding for operationalizing similar grants.

## 5 Conclusions

The present perspective emphasized that the management and implementation of a research grant are crucial for getting the desired outcome. The learnings of PHRI grant execution allow the researchers to understand the issues in terms of methodological rigour and financial guidelines; rigorous tracking of the project activities and complying with the terms of the funding agreement are crucial. These learnings would be helpful to the grant managers and the fellows for effective planning. Improving research management requires a collaborative approach across the institutions and with all managerial levels. The challenges explored in this grant execution recommend developing a structured public health grant management leadership program for the executors and researchers in the future.

## Ethical concerns

The present study does not use confidential data. The permission was obtained within the grant approval that all the information collected during the PHRI grant execution will be used further for research and development. There was a mandate/ instruction from the TAG to document the issues and challenges.Therefore, no additional consent was obtained for this particular perspective.

## Declaration of Competing Interest

The authors declare the following financial interests/personal relationships which may be considered as potential competing interests:

Deepak Saxena reports financial support was provided by Department of Science and Technology.
